# Open reduction versus closed reduction in internal fixation of displaced femoral neck fracture in children: a systematic review and meta-analysis

**DOI:** 10.1186/s13018-023-03525-x

**Published:** 2023-01-17

**Authors:** Eic Ju Lim, Boo-Seop Kim, Minboo Kim, Hyun-Chul Shon, Chul-Ho Kim

**Affiliations:** 1 Department of Orthopaedic Surgery, Chungbuk National University Hospital, Chungbuk National University College of Medicine, Cheongju, Republic of Korea; 2 Department of Orthopaedic Surgery, Hyundae General Hospital, Chung-Ang University College of Medicine, Namyangju-Si, Kyunggi-Do Republic of Korea; 3 Department of Orthopaedic Surgery, Asan Medical Center, University of Ulsan College of Medicine, 88 Olympic-Ro 43-Gil, Songpa-Gu, Seoul, Republic of Korea; 4 Department of Orthopaedic Surgery, Chung-Ang University Hospital, Chung-Ang University College of Medicine, Seoul, Republic of Korea

**Keywords:** Open reduction, Closed reduction, Children, Pediatric, Femoral neck fracture

## Abstract

**Background:**

The quality of reduction is an important factor affecting clinical outcomes for displaced femoral neck fractures (FNFs). However, concerns remain about the invasiveness of open reduction and internal fixation (ORIF) as compared to that of closed reduction and internal fixation (CRIF), and the choice between ORIF and CRIF as an optimal treatment strategy for displaced pediatric FNF remains controversial.

**Materials and Methods:**

MEDLINE, Embase, and the Cochrane Library were systematically searched for studies published up to December 22, 2022, that compared ORIF and CRIF techniques for treating FNF in children. Pooled analysis identified differences in surgical outcomes between ORIF and CRIF, especially regarding postoperative complications, such as osteonecrosis of the femoral head (ONFH), nonunion, coxa vara deformity, leg-length discrepancy LLD, and premature physeal closure (PPC).

**Results:**

We included 15 studies with 635 pediatric FNF cases in our review. Of these, 324 and 311 were treated with ORIF and CRIF, respectively. The pooled analysis revealed that no significant differences existed between each reduction technique for ONFH (odds ratio [OR] = 0.89; 95% confidence interval [CI] 0.51–1.56; *P* = 0.69), nonunion (OR = 0.51; 95% CI 0.18–1.47; *P* = 0.21), coxa vara deformity (OR = 0.58; 95% CI 0.20–1.72; *P* = 0.33), LLD (OR = 0.57; 95% CI 0.18–1.82; *P* = 0.35), and PPC (OR = 0.72; 95% CI 0.11–4.92; *P* = 0.74).

**Conclusions:**

Despite concerns about the invasiveness of ORIF, no differences in complications exist between ORIF and CRIF after FNF in children. Therefore, we believe that ORIF should be performed in FNF when the fracture is irreducible by closed manner.

## Introduction

Displaced femoral neck fracture (FNF) is a rare condition in children that represents a surgical challenge because of the prevalence of severe complications [1]. Pediatric FNF is associated with osteonecrosis of the femoral head (ONFH); nonunion; and high rates of coxa vara deformity, lower limb length discrepancy (LLD), and premature physeal closure (PPC) [2, 3].

Several studies have suggested that the quality of reduction is the most important surgeon-mediated factor affecting the clinical outcomes of displaced FNF in non-elderly patients with trauma [4, 5]. Open reduction and internal fixation (ORIF) may be needed to achieve better fracture reduction, reducing nonunion or angular deformities. The decision to perform ORIF or closed reduction and internal fixation (CRIF), both of which have pros and cons, may be influenced by many factors, including the fracture pattern, patient characteristics, and surgeon preference [6]. Su et al. [7] reported specific fracture patterns of irreducible FNF and recommended a more invasive approach in these cases. In addition, multiple attempts at closed reduction are not encouraged because they can aggravate soft tissue injury and damage the blood supply.

However, concerns exist regarding the risk of open reduction as compared to that of closed reduction. The ORIF of FNF is a more invasive procedure than CRIF because the fragile vasculature of the femoral head could lead to a high risk of vascular injury. In childhood, the epiphyseal plate acts as an absolute barrier to blood flow between the epiphysis and metaphysis, resulting in a more vulnerable femur neck [8]. Several studies have compared ORIF with CRIF to determine the utility and safety of using open reduction to achieve acceptable reduction, optimal internal fixation, and successful outcomes without late sequelae; however, the best option has not yet been established. Papalia et al. [9] concluded that no conclusion could be drawn due to low methodological quality in their review. Clinically, there are still situations where it is difficult to choose between ORIF and CRIF.

Recently, a meta-analysis of case series [10] and a randomized controlled trial [11] that compared ORIF to CRIF in adult FNF were published; however, to the best of our knowledge, no large-scale study or meta-analysis of FNF in children has been conducted. Thus, in this meta-analysis, we compared ORIF and CRIF techniques in pediatric FNFs, with a focus on postoperative complications.

## Materials and methods

This study was performed in accordance with the guidelines of the revised assessment of multiple systematic reviews (R-AMSTAR) and preferred reporting items for systematic reviews and meta-analyses 2020 statement (PRISMA 2020) [12, 13].

### Literature search

MEDLINE, Embase, and Cochrane Library databases were systematically searched for studies that compared ORIF and CRIF for displaced FNF in children up to December 22, 2022. We used search terms in the title, abstract, MeSH, and keywords fields included synonyms and terms related to ORIF, CRIF, FNF, and children, as follows: “open” AND “closed” AND [(“Fracture*”) AND (“femur neck” OR “femoral neck” OR “intracapsular”)] AND (“pediatric” OR “child*”). There were no restrictions on language. After the initial online search, relevant articles and bibliographies were manually reviewed.

### Study selection

From the titles and abstracts, two reviewers independently selected the studies for full-text review. If data from the abstract did not provide enough data to make a decision, the full text of the article was reviewed.

Studies were included in the systematic review if they (1) directly compared ORIF and CRIF techniques for the treatment of FNF, (2) were performed only in children, and (3) were performed for acute traumatic fracture and did not include other fracture types, such as neglected or pathological fractures. We excluded studies if they did not perform internal fixation following fracture reduction (e.g., hip spica cast application after closed reduction); (2) did not report outcomes that would provide us to analyze comparative data; and (4) were duplicate articles from the same research group.

### Data extraction

To synthesize qualitative data, we extracted the following information and variables using a standardized form: (1) study design, (2) number of patients, (3) mean patient age, (4) patient sex, (5) reason for ORIF, (6) follow-up period, (7) fracture type (Delbet classification) [14], (8) delay from injury to surgery, and (9) type of implants used. In the pooled analysis, we extracted the following data from the studies for both the ORIF and CRIF groups: (1) incidence of ONFH, (2) rate of nonunion, (3) incidence of coxa vara deformity, (4) incidence of LLD, and (5) incidence of PPC.

For data extraction, the same two board-certified orthopedic surgeons who selected the studies recorded data independently for each enrolled study. During the screening process, disagreements were resolved through discussion.

### Methodological quality assessment

We used the Methodological Index for Non-randomized Studies (MINORS) [15], which is a tool for assessing the quality of non-randomized studies to assess the methodological quality of the included studies. The maximum MINORS score was 24. Two reviewers assessed the methodological quality and resolved any differences by discussion.

### Data synthesis and statistical analyses

The main outcome of this meta-analysis was the comparison of postoperative complications, including ONFH, nonunion, coxa vara deformity, LLD, and PPC. For all comparisons, we calculated odds ratios (ORs) and 95% confidence intervals (CIs) as dichotomous data. We assessed heterogeneity using the I^2^ statistic, where 25%, 50%, and 75% represented low, moderate, and high heterogeneity, respectively. We used forest plots to show the outcomes, pooled estimates of effects, and overall summary effect of each study. Statistical significance was set at *P* < 0.05. We pooled all data using a random-effects model to avoid overestimation of the study results [16]. We tested for publication bias only with regard to the incidence of ONFH, because publication bias is typically evaluated when at least ten studies are included in a meta-analysis [17]. We performed statistical analyses using Review Manager (version 5.3; Copenhagen, Nordic Cochrane Center, Cochrane Collaboration 2014) and the “Metafor” package in R (version 3.4.3; R Foundation for Statistical Computing, Vienna).

## Results

### Study identification

**Fig. 1:**
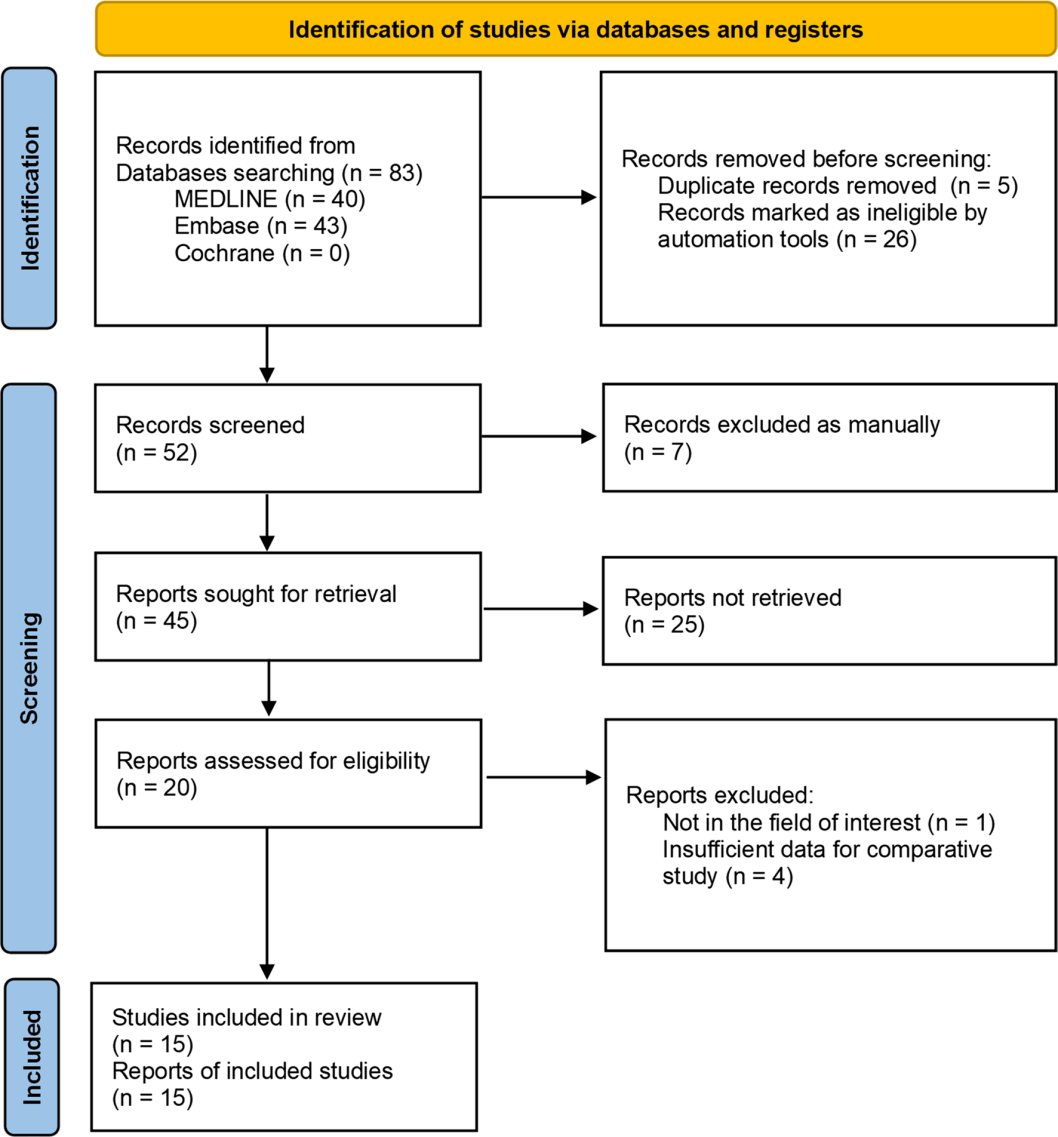
Preferred Reporting Items for Systematic reviews and Meta-Analyses (PRISMA) flow diagram for the identification and selection of studies included in this meta-analysis

Details of our study identification and selection processes are summarized in Fig. 1. An electronic literature search yielded 83 articles. After removing before screening as duplicates and ineligible records following automated tool, we screened the remaining 52 studies. Of these, 32 were excluded after screening their titles and abstracts and five after completing a full-text review. Thus, 15 studies [3, 18–31] were eligible for qualitative and quantitative data synthesis.

### Study characteristics

All 15 included studies had retrospective designs. In total, 635 pediatric FNF cases were included. Of them, 324 patients were treated using ORIF and 311 patients were treated using CRIF. The mean patient age ranged from 8.0 to 12.9 years. With the exception of one study [26], more than half of the patients in the studies were male. Six studies presented the reason for ORIF as failure of CRIF [20, 21, 23, 24, 27, 29], three studies determined the reduction type according to the patient profile and fracture pattern [3, 19, 30], and one study chose the reduction type according to the study period [26]. Postoperative follow-up periods ranged from 2.0 to 6.8 years. All studies included Delbet type II or III as the most common fracture type. Except for three studies that did not include details [19, 22, 25], four studies performed both ORIF and CRIF within 24 h after injury in two-thirds of all patients included [20, 23, 26, 27], and others described the mean surgery delay, which ranged from 1.5 to 11.2 days [3, 18, 21, 24, 28–31]. In these studies, multiple-pin or Kirschner wire fixation, cancellous cannulated screws, or fixed-angled devices were used for the fixation of the FNF. The details of each variable are described in Table 1.

**Table 1:** Study design, demographic data, study characteristics and MINORS scores for studies included in the meta-analysis

References	Study design	No. of patients		Age (range) (years)	Male sex (%)	Reason for ORIF	F/U period (years)	Delbet classification	Surgery delay (range)	Implant used	MINORS score
		ORIF	CRIF								
Azam et al. [18]	RCS	8	14	9.0 (5−15)	63.6	N/A	> 2	II: 59%, III: 41%	11.2 days (2−21)	N/A	19
Bali et al. [19]	RCS	18	13	10.0 (3−16)	55.6	Patient profile, fracture pattern	3.2	II: 48.4%, III: 25.8%, IV: 25.8%	N/A	CCS, FAD	19
Cheng et al. [20]	RCS	3	7	12.9 (9−16)	70.0	Failure of CR	4.6	I: 7%, II: 57%, III: 29%, IV: 7%	< 24 h	CCS, FAD	18
Dai et al. [30]	RCS	31	13	9.0 (2−14)	61.0	Patient profile, fracture pattern	4.8	I: 2%, II: 57%, III: 41%	4.3 days (1−19)	CCS, FAD	19
Dendane et al. [21]	RCS	13	8	12.1 (5−16)	66.7	Failure of CR	2.2	II: 42.9%, III: 47.6%, IV: 9.5%	4.9 days (0.5−21)	Pin, CCS, FAD	17
Dhammi et al. [22]	RCS	9	17	10.8 (3−17)	53.8	Failure of CR	2	II: 38.5%, III: 61.5%	N/A	Pin, CCS	17
Inan et al. [23]	RCS	5	34	11.1 (4−16)	56.4	Failure of CR	3.4	II: 53.8%, III: 35.9%, IV: 10.3%	≤ 24 h: 26, > 24 h: 13	K-wire, CCS, FAD	18
Ju et al. [24]	RCS	37	21	9.1 (1.7−15.6)	69.0	Failure of CR	2.9	II: 51.7%, III: 36.2%, IV: 12.1%	4.7 days (4.29 CR, 4.89 OR)	K-wire, CCS, FAD	17
Lin et al. [25]	RCS	19	15	8.0	58.8	N/A	1 − 3	II: 73.5%, III: 26.5%	N/A	K-wire, CCS	17
Moon et al. [31]	RCS	6	16	8 (1.5−16)	50.0	N/A	4.0	II: 32%, III: 40%, IV: 28%	1.5 days	Pin, CCS	19
Song et al. [26]	RCS	15	12	9.9 (5−16)	40.7	Period	2.8	II: 55.6%, III: 44.4%	≤ 24 h: 24, > 24 h: 3	Pin, K-wire, CCS, FAD	19
Stone et al. [27]	RCS	6	16	11.5 (4.5−17.4)	59.1	N/A	2.1	II: 59.1%, III: 36.4%, IV: 4.5%	≤ 24 h: 18, > 24 h: 4	K-wire, CCS, FAD	20
Varshney et al. [28]	RCS	8	13	11.8 (5−15)	66.6	Failure of CR	6.8	II: 66.6%, III: 33.3%	8.1 days (2−22)	CCS	17
Wang et al. [29]	RCS	138	103	10.0 (2−17)	56.9	N/A	1.6	I: 2.1%, II: 67.6%, III: 29.9%, IV: 0.4%	3.7 days	K-wire, CCS, FAD	17
Wu et al. [3]	RCS	8	9	10.4 (1−14)	58.8	Patient profile, fracture pattern	1.9	I: 12.5%, II: 68.8%, III: 18.8%	3.5 days (1.1−10.0)	K-wire, CCS	19

*CCS* cancellous cannulated screws, *CR* closed reduction, *CRIF* closed reduction and internal fixation, *FAD* fixed-angled device, *MINORS* Methodological Index for Nonrandomized Studies, *N/A* not available, *ORIF* open reduction and internal fixation, *RCS* retrospective comparative study

### Risk-of-bias assessment

The mean MINORS score for methodological quality assessment was 18.1/24 (range, 17–20) (Table 1). Five studies received a point deduction for the lack of description of consecutive inclusion [21, 22, 24, 25, 29]. All 15 studies received a point deduction due to their retrospective study design and lack of double-blind evaluation for subject endpoints. Four studies received a point deduction for loss to follow-up of up to 5% of patients compared with the initial number of patients enrolled [20, 23, 27, 28]. All 15 studies received a point deduction in the study size calculation domain as none of these studies prospectively calculated the sample size. There were no point deductions for any other criteria.

### Quantitative data synthesis

**Fig. 2:**
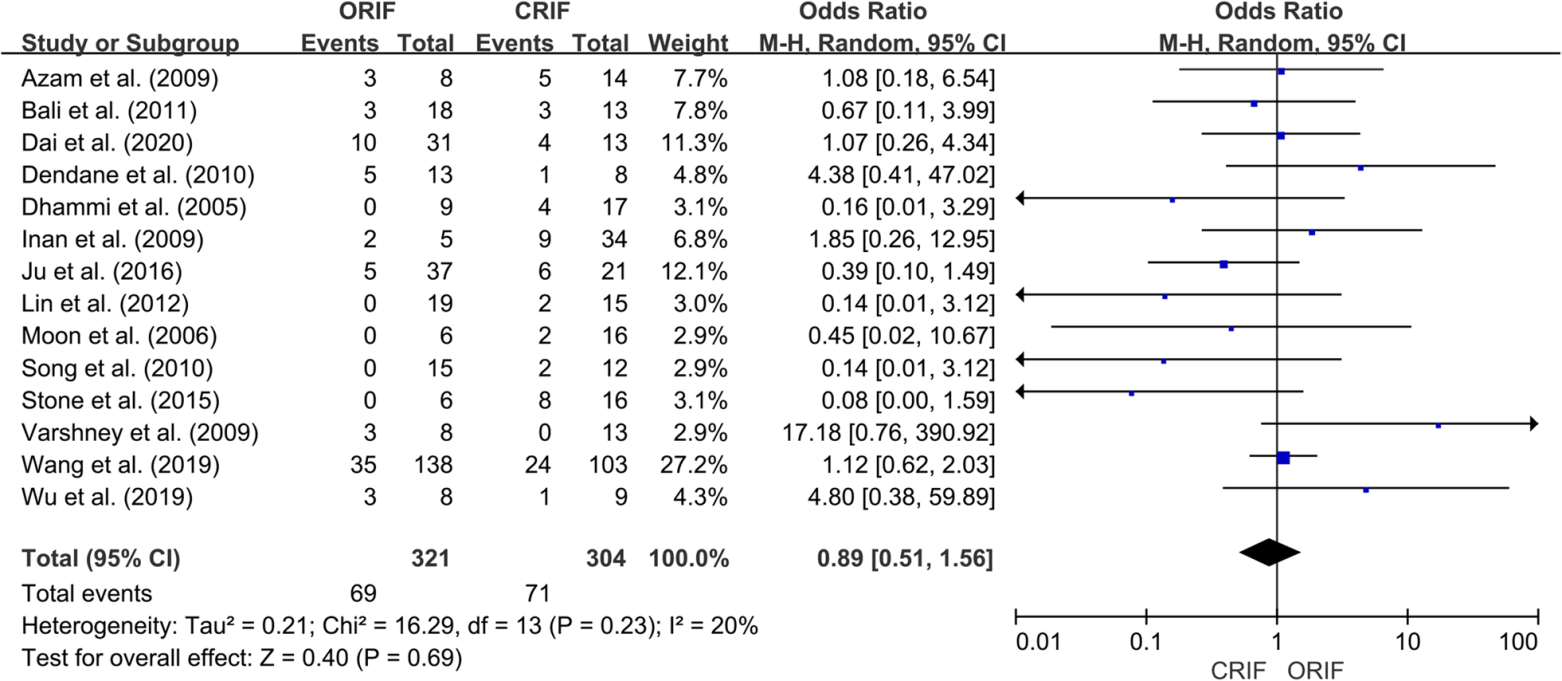
Forest plot showing the incidence of ONFH after pediatric FNF between the ORIF and CRIF groups

**Fig. 3:**
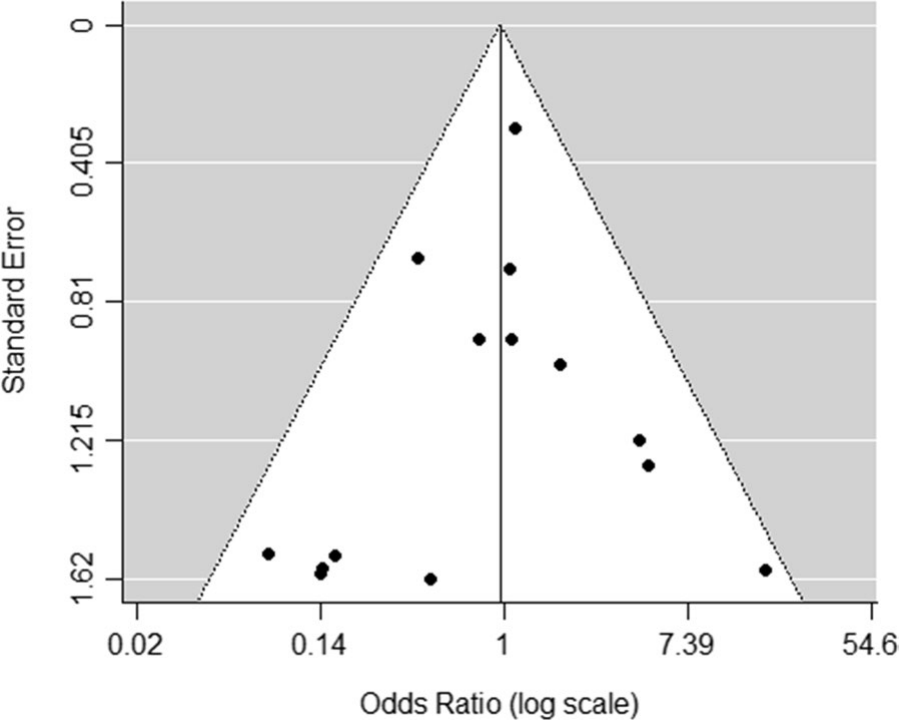
Funnel plots showing no publication bias for the incidence of ONFH

**Fig. 4:**
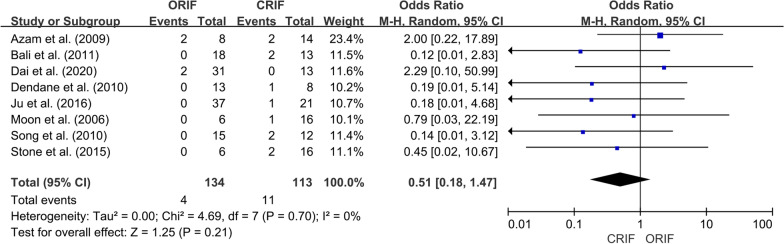
Forest plot showing the nonunion rate after pediatric FNF between the ORIF and CRIF groups

**Fig. 5:**
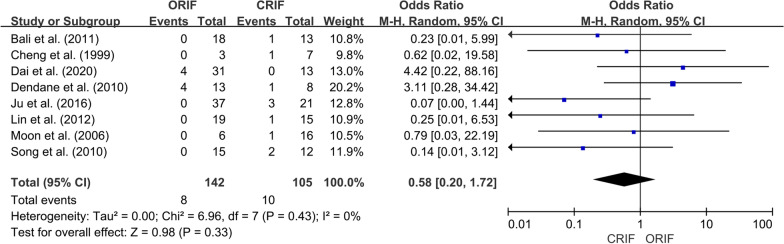
Forest plot showing the incidence of coxa vara deformity after pediatric FNF between the ORIF and CRIF groups

**Fig. 6:**
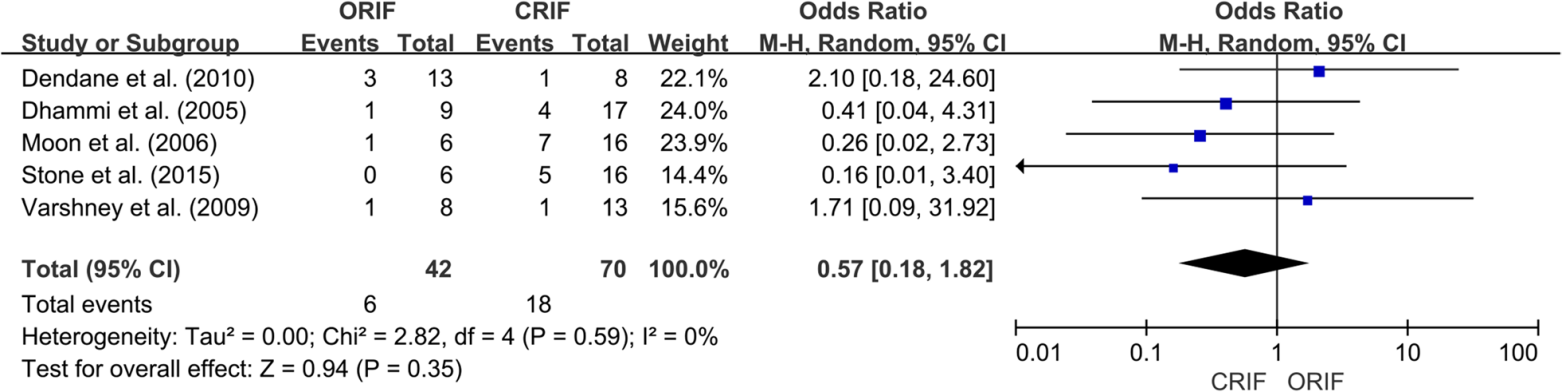
Forest plot showing the incidence of LLD after pediatric FNF between the ORIF and CRIF groups

**Fig. 7:**
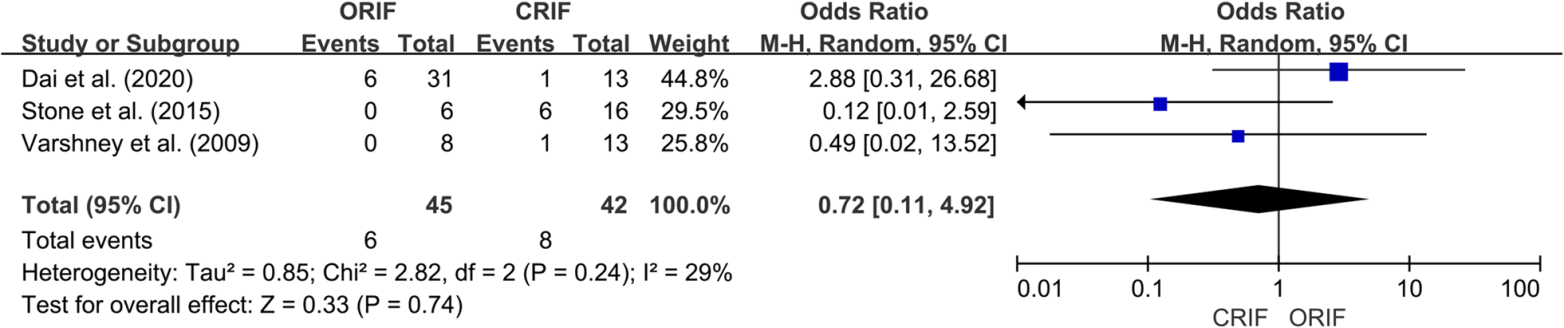
Forest plot showing the incidence of PPC after pediatric FNF between the ORIF and CRIF groups

1. *Incidence of ONFH*
  We extracted data regarding the number of cases of ONFH in both the ORIF and CRIF groups from 14 of the 15 studies [3, 18, 19, 21–31]. Postoperative ONFH was reported in 69 of 321 patients in the ORIF group and 71 of 304 patients in the CRIF group. The pooled analysis showed no differences in the incidence of ONFH between the two groups (OR = 0.89; 95% CI 0.51–1.56; *P* = 0.69). Heterogeneity was considered low (*I*^2^ = 20%). Forest plots and details are shown in Fig. 2. Egger’s test showed no publication bias existed following visual assessment of the funnel plot (Fig. 3) regarding the incidence of ONFH (*P* value for the test of funnel plot asymmetry = 0.46).
2. *Non-union rate*
  Data on nonunion rates following ORIF and CRIF treatment for pediatric FNF were extracted from eight studies [18, 19, 21, 24, 26, 27, 30, 31]. Nonunion was reported in 4 of 134 cases in the ORIF group and 11 of 113 cases in the CRIF group. The pooled analysis revealed that the nonunion rate did not differ significantly between ORIF and CRIF (OR = 0.51; 95% CI 0.18–1.47; *P* = 0.21). Heterogeneity was considered low (*I*^2^ = 0%). Forest plots and details are shown in Fig. 4.
3. *Coxa vara deformities*
  Eight studies [19–21, 24–26, 30, 31] compared the incidence of coxa vara deformity following pediatric FNF between ORIF and CRIF. This deformity was reported in 8 of 142 patients in the ORIF group and 10 of 105 patients in the CRIF group. The pooled analysis showed no difference in the incidence of coxa vara deformity between the two groups (OR = 0.58; 95% CI 0.20–1.72; *P* = 0.33). Heterogeneity was considered low (*I*^2^ = 0%). Forest plots and details are shown in Fig. 5.
4. *Incidence of LLD*
  We extracted data on the incidence of postoperative LLD in five studies [21, 22, 27, 28, 31]. Postoperative LLD was reported in 6 of 42 patients in the ORIF group and 18 of 70 patients in the CRIF group. The pooled analysis showed no differences in the incidence of LLD between the two groups (OR = 0.57; 95% CI 0.18–1.82; *P* = 0.35). Heterogeneity was considered low (*I*^2^ = 0%). Forest plots and details are shown in Fig. 6.
5. *Incidence of PPC*
  We extracted data on the incidence of PPC in three studies [27, 28, 30]. PPC was reported in 6 of 45 patients in the ORIF group and 8 of 42 patients in the CRIF group. The pooled analysis showed no differences in the incidence of PPC between the two groups (OR = 0.72; 95% CI 0.11–4.92; *P* = 0.74). Heterogeneity was considered low (*I*^2^ = 29%). Forest plots and details are shown in Fig. 7.

## Discussion

The principal finding of this pooled analysis was that there were no apparent differences in the incidence of postoperative complications after pediatric FNF between the ORIF and CRIF groups, especially for ONFH, nonunion, coxa vara deformity, LLD, and PPC. Given the consensus that the quality of reduction is the most consistent predictor of successful treatment for displaced FNF [4, 5], we believe that ORIF should be performed in FNF when it is required for anatomical reduction, especially on the fracture irreducible by closed manner.

A lack of randomization exists for the use of open reduction, raising concerns about the effects of bias in previous studies. In addition, in the present synthetic analysis, 8 of 15 studies presented that the decision to use ORIF was determined by whether FNF was reducible using the closed method. As ORIF is performed for fractures that fail CRIF, these fractures may have been more displaced, or more difficult fractures to begin with. Therefore, we cannot interpret the finding that no significant difference exists between ORIF and CRIF.

ONFH in children is the most common complication following FNF, with a reported incidence of 0–92% in the literature [32]. Wang et al. [29] reported that age and initial displacement were independent predictors of ONFH in pediatric FNF and insisted that, to avoid ONFH, adequate reduction is more important than the type of reduction. Upadhyay et al. [11] demonstrated that no significant differences existed in the quality of reduction and ONFH between the ORIF and CRIF groups in their prospective, randomized study. However, they excluded four patients in whom an acceptable closed reduction could not be obtained, and these patients were managed using open reduction. Song studied ORIF not by the fracture pattern but by the study period, where CRIF was performed regardless of the fracture pattern during the “CRIF period” [26]. Under these conditions, Song reported that CRIF without considering the fracture pattern demonstrated poor reduction and more complications, including ONFH.

Nonunion in pediatric FNF is much less common than in adult FNF because of the presence of a thick functional periosteum, but once nonunion develops, it is associated with poor patient outcomes [33]. Including very recent study from Wang et al., initial displacement, delayed surgery, inadequate reduction status, and poor fixation are the major causes of nonunion [34–36]. Ju et al. [24] concluded anatomical reduction must be achieved after FNF to reduce the incidence of nonunion, regardless of whether CRIF or ORIF is used. In their meta-analysis, Yeranosian et al. [37] suggested that the approach to the treatment of pediatric FNF should become more interventional because of the high complication rate with unsatisfactory reduction over the years.

Consistent emphasis has been placed on anatomical reduction in both ONFH and nonunion. Although no significant differences were found between ORIF and CRIF for both ONFH and nonunion in the present study, the outcomes of the ORIF group were confounded by the large number of more severe fractures. We believe that it is important to achieve anatomical reduction regardless of the reduction type.

In the current study, the incidence of coxa vara deformity, LLD, and PPC also showed no differences between the ORIF and CRIF groups. Although these conditions may occur alone or in combination [21, 28, 32], the consensus is that the incidence of coxa vara deformity is related to reduction status, which means that its likelihood could be diminished with anatomical reduction and internal fixation [34, 38]. Although our current synthetic results did not reveal the superiority of ORIF over CRIF with respect to complications, theoretically, ORIF is more beneficial than not in treating FNF because it is required to avoid an unacceptable reduction status, which can lead to serious complications.

Specific fracture patterns of irreducible FNF have been reported, and a more invasive approach has been recommended for these types of FNF [7]. However, all included studies classified fractures according to the Delbet classification system, which divides fractures according to their location [14]. Using this classification, it is difficult to determine whether a satisfactory reduction is possible using only CRIF. Before multiple attempts at closed reduction, it is better to identify which fracture patterns are impossible to repair with CRIF and make an appropriate decision. Therefore, further studies are required to confirm this hypothesis.

The current meta-analysis has several limitations. First, although a satisfactory number of studies were included, all were retrospective in nature. Pooling the results of predominantly retrospective studies may overestimate the outcomes. Nevertheless, considering that our study is the first meta-analysis to provide a general overview of this topic and no publication bias was observed, our synthetic results are meaningful. Second, as limited data were available, we could only conduct the meta-analysis for all variables as dichotomous data and not as continuous data, especially for the degree of coxa vara deformity or LLD. Although the current study showed no significant differences in these variables between the types of reduction, converting these variables to continuous data may make a statistically significant difference. Third, owing to the characteristics of the meta-analysis, we could not control for fracture severity as a confounding factor, even with our best efforts to reduce the bias from each included study. Therefore, we need further high quality studies to analyze these issues more clearly.

## Conclusion

Despite concerns about the invasiveness of ORIF, no differences in complications exist between ORIF and CRIF after FNF in children. Therefore, we believe that ORIF should be performed in FNF when the fracture is irreducible by closed manner.

## Data Availability

The datasets used and/or analyzed during the current study are available from the corresponding author on reasonable request.

## Abbreviations

FNF: Femoral neck fracture
ONFH: Osteonecrosis of the femoral head
LLD: Limb length discrepancy
PPC: Premature physeal closure
ORIF: Open reduction and internal fixation
CRIF: Closed reduction and internal fixation
MINORS: Methodological index for non-randomized studies
ORs: Odds ratios
CIs: Confidence intervals
